# Supplementary figures and images for: Benchmark Dataset for Training Machine Learning Models to Predict the Pathway Involvement of Metabolites
Source: Metabolites. 2023 Nov 1;13(11):1120. doi: 10.3390/metabo13111120 (PMC10673125; doi:10.3390/metabo13111120)

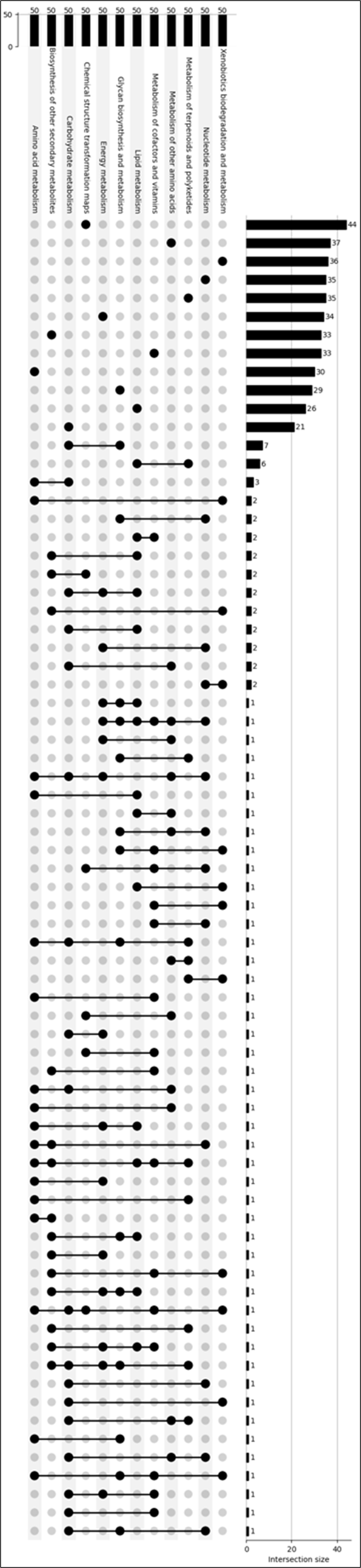

Supplement: Supplementary file 1 [file metabolites-13-01120-s001.zip › Figure S3 – Upset plot showing overlap between pathway categories of their top 50 most important features..png]
